# Veratrum Viride

**Published:** 1860-11

**Authors:** E. Platt


### SELECTIONS.

### *Veratrum Viride.*

In the April number of the *Gazette* for 1859, is an article which I furnished, on the subject of Veratrum Viride, giving the result of my experience with that very active medicament, for the previous two years, and a number of cases, showing that its most striking and noticeable effects on the human organism were those of a pure sedative of great energy, which are sometimes manifested with extreme suddenness, producing very unpleasant, and even dangerous results; and although I admitted that it might be used in inflammatory and dynamic diseases in such a manner as to obtain from it beneficial results, yet that I had never witnessed *in any case* those remarkably good effects that some physicians claim to have done. I likewise, in the same number, made some remarks on an article of Dr. Close, of Port Chester, "On the Bradycrote or Abortive Treatment of Fevers," by veratrum, digitalis and aconite, in which the doses recommended by him seemed to me dangerously large; referring particularly to a case stated by him, of a child between three and four years old, for which he had prescribed tinct. veratrum, gtt. 3; digitalis, gtt. 5; aconite, gtt. 2; to be repeated every two hours, and which was thus continued for three or four days, with, in this instance, apparently good effects, as the child recovered. As a counterpart to this, and to show the uncertainty of the action of veratrum on the system, I related the case of a little girl *ten years old* who had acute pneumonia, who by *one dose only*, of three drops of the saturated tinct., came very near being killed; in which case there could be no doubt as to the effects being due to the veratrum, as the pulse was reduced from over 100 to 60 per minute.

Dr. Close replied to these remarks, and assured us that he had used veratrum, combined with digitalis and aconite, for eight or ten years, *without one unpleasant result, in any case, during the whole of that time;* and that he had almost uniformly succeeded in cutting short in a very few days (from 3 to 8) "*fevers*" of every description, without reference to their cause. Altough it appeared to me that Dr. Close used the term "*fevers*" in a sense somewhat vague, not stating explicitly whether he intended to include under it original, idiopathic fevers only, or all diseases which were attended with fever, still, in either case, his success had been so remarkable, that I resolved to make further observations and trials with veratrum and digitalis, before making any reply on the subject; and this I have done from that time to the present, and am now obliged to say, that these numerous further experiments have confirmed me in the correctness of the opinions expressed in my former communication on this subject; and all I have to say as regards Dr. Close's statement is, that if he or others succeed, as a general rule, in arresting *fevers* of every description, by means of veratrum, with or without digitalis and aconite, never encountering any unpleasant consequences, I rejoice at their success, freely confessing that I have not been able to obtain like happy results.

Since my previous report, I have used veratrum with much more caution than before, in smaller doses, and generally combined with digitalis and morphine, as recommended by Dr. Close, with the view of avoiding, if possible, its nauseating, and sometimes very suddenly depressing effects; and I may add that this plan, so far as these upleasant effect are concerned, has in the main been successful; but not entirely so, for a few instances have occurred in which they manifested themselves very suddenly, to the great alarm of the friends of the patients. The following is one of them, which occurred last November, in a stout, able-bodied farmer, who had a severe attack of pleuro-pneumonia. After bleeding to 20 oz., I put him on the use of the following: Veratrum, gtt. 4; digitalis, gtt. 6; solut. morphia, gtt. 3; to be repeated every three hours, thinking that if this prescription was ever indicated, it was so in this case. This was in the evening; on the following day I saw the patient, and ordered the medicine to be continued. Mark the consequence: the succeeding night, at 2 o'clock, I received a message requesting my attendance as speedily as possible, the friends of the patient fearing he would die before morning. This was unlook-for intelligence, as only a few hours previous there was not one alarming symptom. As the night was one of Egyptain darkness, and the patient lived six miles distant, I had, consequently an extra-delightful ride, for which I at once suspected I was indebted to the veratrum. After ascertaining the state of the patient, I was not surprised at the alarm of his friends. He was nearly in a state of collapse; his extremities were cold, he was sweating profusely, he had vomited much, his bowels were very loose, and he complained of a distressing feeling of exhaustion and sinking, and his pulse was reduced from over 100 to 50. This last showed beyond a doubt the cause to which all these disagreeable symptoms were to be ascribed. I must not omit a very striking fact. The cough and pain in the chest, both of which had previously been acute, had ceased entirely, and they did not return under three or four days, and then were comparatively slight. The unpleasant symptoms were relieved by dry, hot applications to the extremities, and an anodyne of solut. morph., with aromatics. In ten days therefrom the patient had entirely recovered. Now it is very probable that, in this case, the powerfully sedative effect of the veratrum and digitalis after the venesections greatly aided, or was the sole cause of the solution of the thoracic inflammation, thus abruptly cutting short the disease. Remedies, however, that should often operate in this violent manner, would, as a general rule, both by patients and their friends, be regarded as altogether too heroic, and belonging to that class that either cure or kill.

Physicians of my acquaintance of whom I have inquired as to the result of their trils of veratrum, all concur, or all who have used it to much extent, in saying that they have had cases similar to that above stated. Most of them have used Tilden & Co's Extract, which, although a good preparation, is much inferior in strength to the saturated tincture; and as they generally prescribed it in about the same doses, four or five drops, this is one reason, or the sole reason, why they do not very frequently occur in their practice. In any case of acute inflammatory disease, it is always desirable, and sometimes of the last importance, to bring the system as speedily as possible under the controlling influence of remedies; and if, in order to do this, we resort to veratrum, or veratrum and digitalis, either with or without bloodletting, and give efficient doses, say 5 or 6 drops every three hours, we may possibly effect, and speedily, our object. Unfortunately, however in every case thus treated, we are compelled to take the risk, unless we have the luck to have but one patient, and can afford to remain with him, of its producing, at very short notice, great and perhaps dangerous prostration, thereby greatly alarming the patient and his friends, and giving us the change of receiving, perhaps in the middle of the night, the pleasant message that our patient is supposed to be dying.

On the other hand, giving in doses of 2 or 3 drops, either with or without 5 or 6 drops of digitalis, it may generally be continued for some time, a week or two, with probaly no sensible effect, except, perhaps, that the disease for which it was prescribed gradually declines; and it might have done so without it; but from its unquestionable sedative powers, the inference is rational that in an inflammatory disease, it may, in these small doses, have contributed to that end. Its effects, however, in this way, are merely presumptive or inferential; by no means positive or certain. Dr. Norwood, of Charleston, is said to have employed it not only in pulmonary inflammations, but also in Typhoid Fever. Of its effect in this disease I can say nothing from my own observations, for I should as soon think of putting a lancet in to the patient's arm, as of prescribing veratrum; and if Dr. Norwood, in the treatment of this disease with this sedative, met with the success that is claimed, I am entirely satisfied that his patients recovered in spite of his remedy, which received the credit due to nature alone; a circumstance, perhaps, not very uncommon. If it had any curative effect in in this disease, it could be on no other than the homœopathic principle of "*Similia*, &c.;" and as we all know this *last* to be a fiction, there is in my mind no doubt but that the first is also. Our profession formerly was so overlaiden with false facts and hasty generalizations, leading of course to false theories, and such contrariety of practice, as almost to justify the sarcasm that in our profession "*nothing but certainty is certain.*" Within the last few years, however, many medical cobwebs have been brushed away, and there are some remaining that require, and will certainly receive, the same treatment.

Dr. Norwood's doses of veratrum were 8 drops of the saturated tinct., every three hours increasing one drop at a time until the pulse was reduced, or nausea and vomiting ensued; which last, I think, would soon follow, and then, as a consequence, very probably the first. Dr. Wood, in the *U. S. Dispensatory*, says, "6 to 8 drops, every three hours, and increased to 8 if necessary, until its effect are produced, may be given with safety." He adds, however, that it should be given "with caution." He should have said, *very great caution.* The powerfully depressing effect produced upon a strong man, laboring under an inflammatory disease, by 3 drops with 6 drops of digitalis every three hours, is shown in the case given above in this paper; and in the April number of the *Gazette*, for 1859, I reported six cases, the subjects of two of which *barely escaped with their lives;* of this any one who will take the trouble to turn to those cases will, I think, be convinced. In none of these did the doses exceed five drops every three hours.

Like causes must produce like effects, and those physicians who have an extensive practice, and frequently prescribe veratrum, in Dr. Norwood's and Dr. Wood's doses, must meet with cases similar to those I have reported, unless they remain constantly with their patients, and carefully watch its effects, which few can afford to do. *This is the only safe method of using it in those doses.* Those who do not do this, and yet meet with none such, use most probably a preparation of inferior strength, as I am perfectly confident, from my own observations, that the peculiar susceptibility to the depressing action of veratrum, that idioyncrasy that can neither be foreknown nor explained, exists in so many persons, that it is all but impossible that otherwise they could have been entirely avoided.—*American Med. Gazette.*

E. Platt.

